# Preoperative HAIC enlarges future liver remnant in initial unresectable HCC patients with Vp3 tumor thrombus

**DOI:** 10.3389/fonc.2026.1906245

**Published:** 2026-10-05

**Authors:** Yifan Zhang, Yiquan Lu, Yuchen Yang, Xinyi Luo, Nan Wang, Fengjie Hao, Weixia Li, Yongjun Chen, Junqing Wang

**Affiliations:** 1 Department of General Surgery, Ruijin Hospital, Shanghai Jiao Tong University School of Medicine, Shanghai, China; 2 Department of Internal Medicine III, University Hospital Rheinisch-Westfälische Technische Hochschule Aachen, Aachen, Germany; 3 Department of Radiology, Ruijin Hospital, Shanghai Jiao Tong University School of Medicine, Shanghai, China

**Keywords:** conversion therapy, FLR enlargement, HAIC, hepatocellular carcinoma, VP3

## Abstract

**Background:**

Conversion strategy is the optimal treatment for patients with unresectable hepatocellular carcinoma (uHCC). However, a severe lack of future liver remnant (FLR) may result in early withdrawal. In this small sample retrospective study, we investigated FLR changes after conversion therapies. Notably, patients with ipsilateral Vp3 portal vein tumor thrombus (PVTT) showed a significant FLR increase after treatment and provided an advantage for carrying out radical surgery.

**Methods:**

53 uHCC patients with Vp0 to Vp3 tumor thrombus were enrolled and divided into two groups (Vp0–2 and Vp3) based on the initial PVTT status. All patients received anti-PD-L1 and anti-VEGF therapy combined with hepatic arterial infusion chemotherapy (HAIC) every three weeks. The FLR was measured at baseline and after 3–4 cycles. The odds ratio (OR) between Vp3 and FLR changes was calculated.

**Results:**

Among the 53 patients, 28 achieved complete response (CR) or partial response (PR) after conversion treatment. FLR did not change in Vp0–2 group, regardless of the response. While a significant FLR increase was observed in Vp3 group, especially in patients with CR and PR. 10 cases of successfully converted Vp3 patients showed an average FLR enlargement of 18.5%.

**Conclusion:**

According to our study, Vp3 patients demonstrated greater FLR enlargement after conversion therapy combined with HAIC, probably due to the thrombus’ restriction on blood flow to the tumor side liver. This observation may help to increase both the surgeons’ and the uHCC patients’ confidence in continuing treatment.

## Introduction

Hepatocellular carcinoma (HCC) ranks 3rd globally and is a leading cause of cancer-related deaths because of its aggressive tumor behavior (1). In the real world, advanced HCC is obstinate in treatment, and nearly 10–40% of patients have vascular invasion at the initial diagnosis, which strongly challenges researchers and clinical doctors in setting appropriate treatment strategies (2). Based on most current guidelines worldwide, radical resection is limited and not recommended as the preferred strategy in patients diagnosed with macrovascular invasion or severe portal vein cancer thrombus (PVTT) (3).

Based on the American Association for the Study of Liver Disease/Barcelona Clinic for Liver Cancer (AASLD/BCLC) staging system and treatment guidelines, systemic or conservative treatment is recommended for HCC patients with PVTT, who are regarded at advanced stages. However, the poor outcomes and short survival periods of these patients are disturbing. In Asia, especially in Eastern Asian cases, predominantly Chinese patients, who comprise nearly 50% of new incident cases worldwide, systemic treatments combined with locoregional therapies such as transarterial chemoembolization (TACE) and hepatic arterial infusion chemotherapy (HAIC) have been generally applied as an effective conversion strategy for downstaging advanced HCC (4). According to data, combined therapy has presented higher efficiency in tumor downstaging and provides more opportunities for consequential operative treatment (5). The latest version of the China Liver Cancer Staging (CNLC) system recommends a relatively aggressive conversion therapy for patients from IIb to IIIa stages to convert the advanced stage patients for complete response (CR) pathologically or partial response (PR), meeting the resectable surgical condition (6).

Notably, liver failure is a complication presumably caused by insufficient liver remnant quantity with inadequate hepatocyte function and accounts for 3/4 of the mortality after liver resection for cancer (7). Since it is often too late to diagnose liver failure after surgery, the future liver remnant (FLR) of converted patients is one of the most important issues in determining the safety and prognosis of resection. Generally, in patients with normal liver function, the FLR is recommended to be no less than 30% after surgery, and for patients at risk of liver failure, the remnant liver should be kept above 40% (8). Inadequate FLR has been a great obstacle for HCC patients with underlying liver disease, such as HBV, when considering surgery, even after CR or PR. Such dismal outcomes have discouraged some late-stage HCC patients from continuing standard treatment. In clinical practice, inadequate FLR and compromised liver function are two main factors associated with shorter survival in HCC patients (7).

Theoretically, the existence of PVTT at initial diagnosis, especially for Vp3 cases, which indicates vascular invasion involving the primary branch or main trunk of the portal vein, commonly represents a series of disadvantages in prognosis, including increased risk of tumor spread, higher portal pressure, reduction of portal blood flow, and higher risk of ascites and liver failure (9). However, as we observed, an unexpected and interesting enlargement of the FLR occurred among the patients in group Vp3 (not for Vp4) with significance after the systemic conversion treatment combined with HAIC as the local-regional therapy (LRT), especially with enlargement of the left liver in cases of right portal vein branch invasion. This phenomenon provided partial protection of residual liver function and probably created opportunities for radical surgery. We speculated that PVTT involving the primary branch on the tumor-side hemi liver might induce enlargement of the opposite hemi liver, similar to the effect induced by portal vein embolization (PVE). To prove this hypothesis, we analyzed 53 cases of advanced HCC undergoing conversion therapy during the last three years to analyze the potential factors associated with Vp3 PVTT and FLR enlargement. We believe that our findings may help establish an appropriate and individualized conversion strategy for patients with HCC with severe PVTT.

## Materials and methods

### Study population

Between January 2021 and December 2023, consecutive patients with intermediate-to-advanced HCC who underwent conversion therapy at Hepatobiliary Surgery, Ruijin Hospital, Shanghai Jiao Tong University School of Medicine, were retrospectively screened. Baseline evaluation included liver-enhanced MRI and/or contrast-enhanced CT, contrast-enhanced ultrasound (CEUS) when necessary, serum alpha-fetoprotein (AFP), Protein Induced by Vitamin K Absence or Antagonist-II (PIVKA-II), liver function tests, hepatitis status, Child–Pugh classification, Eastern Cooperative Oncology Group (ECOG) performance status, and tumor staging. Patients were staged according to the CNLC staging system and the BCLC staging system. PVTT was classified using the Japanese Vp classification (10, 11). Eligible patients had Child–Pugh class A or selected class B liver function, ECOG performance status (PS) 0–1, and adequate hematologic and renal function.

A total of 53 patients with Vp0–Vp3 PVTT (CNLC IIb–IIIa) were included in the final analysis. All patients were initially considered unresectable after multidisciplinary team (MDT) evaluation according to one or more of the following criteria: macrovascular invasion, insufficient predicted FLR, extensive tumor burden, multifocal intrahepatic disease, or inability to achieve R0 resection at baseline. Patients with Vp4 tumor thrombus involving the main portal vein trunk or contralateral portal vein branch, and patients with distant metastasis were excluded. First-line systemic therapy (atezolizumab + bevacizumab, atezolizumab + bevacizumab, AB) and HAIC using calcium folinate, fluorouracil and oxaliplatin were conducted according to Chinese guideline (11). AFP, PIVKA-II, liver function tests and tumor imaging including CEUS, MRI and CT were performed regularly to assess treatment effect.

### Conversion therapy strategy

Relevant conversion therapy was administered after collecting the patient’s baseline information. The systemic therapy using the Atezo/Bev program was strictly according to the protocol assigned by ASCO guidelines and the recommendation by Standardization for Diagnosis and Treatment of Primary Hepatic Carcinoma (China) one cycle every 3 weeks. The HAIC program was carried out as the standard LRT in this study along with the AB program in the first three cycles, using a modified FOLFOX regimen recommended by the consensus in China (12). The HAIC-FOLFOX regimen was administered for no more than four cycles.

### Follow-up and FLR calculation

After collecting baseline information, follow-up investigations were also carried out for each patient at baseline and before each therapeutic cycle. These included measurements of AFP and PIVKA-II, and assessment and observation of adverse effects (AEs). Radiological examination was conducted after three cycles using MRI and CT scanning to assess the objective response of the treatment according to the RECIST and mRECIST standards for HCC. The volume of the separated hepatic segments and the predicted FLR were calculated using the SenseCare^®^ Liver Surgical Planning System (SenseTime Health, Shanghai, CN). Briefly, this system is an innovative integration algorithm for artificial intelligence (AI) diagnosis and treatment platforms, and employs deep learning and medical image processing technologies, which can automatically identify, reconstruct, and process radiology images (**Figure 1**). Using this system, we vividly illustrated the lesion’s spatial form, size, location, and relationships with intrahepatic ducts for real-time interactive and quantitative analysis of intrahepatic anatomical structures and a range of resection simulations. By precisely measuring the volumes of the resected parts and the remaining liver, liver reserve function can be efficiently evaluated.

**Figure 1:**
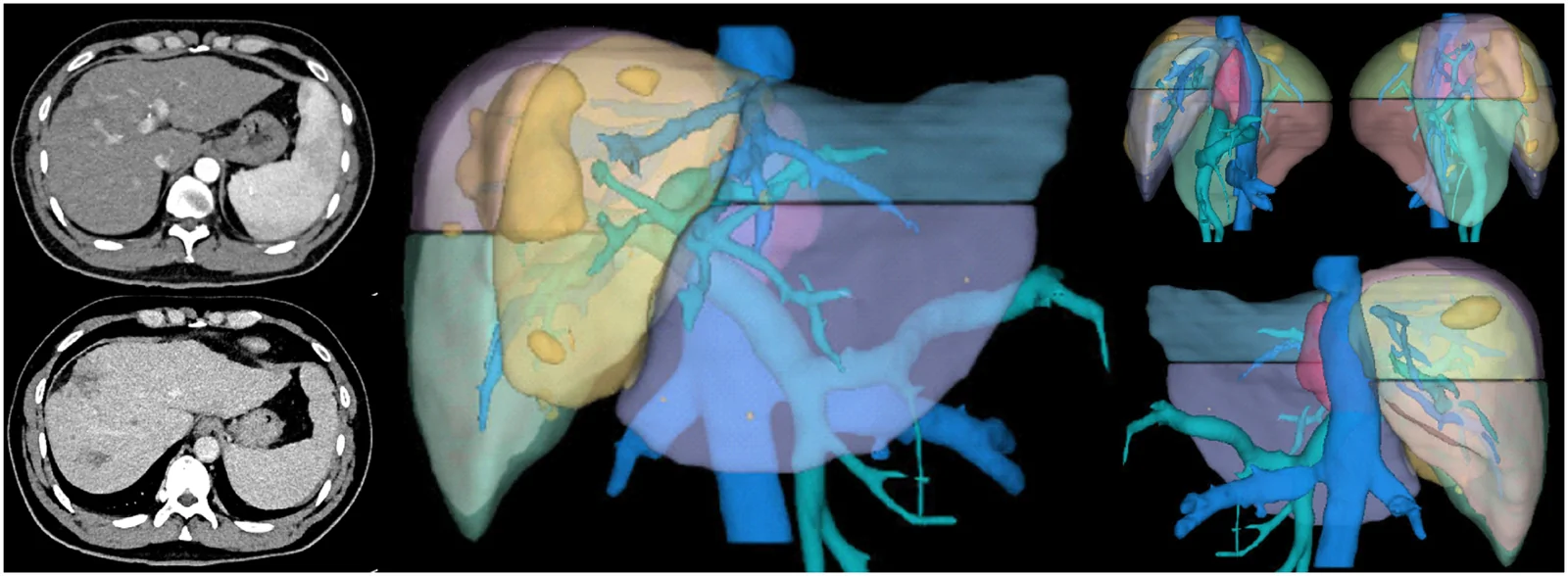
Illustrations of the rebuilding of 3D liver model. Couinaud classification of liver segments was achieved via precise modeling of the portal vein and the inferior vena cava.

### Pathological examination after surgical resection

Patients who presented objective responses after 3–4 cycles were subjected to surgical resection following detailed MDT consultation and evaluation. After the conventional pathological examination, HE-stained slides were prepared from the specimens. Two experienced pathologists reviewed the slides and assessed the necrosis rate of the tumor and the portal vein thrombus.

### Statistical analysis

Statistical analyses were conducted using SPSS 20.0. The FLR change rate (%) was calculated using the formula: (FLR after 3 cycles - baseline FLR) / baseline FLR×100. Spearman correlation analysis was used to describe the correlation between the various parameters and FLR enlargement. *P*-values were calculated using Student’s *t*-test and Fisher’s exact test. For continuous variables, paired and unpaired *t*-tests applied to paired or independent data, respectively. Differences were considered statistically significant at *P*-values < 0.05.

## Results

### Vp3 patients presented a more severe tumor burden at baseline

Among the 53 cases, 44 (83%) were initially diagnosed at the CNLC IIIa stage (**Figure 1A**), which involved vascular invasion, and 86.4% of them presented a relatively severe PVTT status up to Vp3 (**Figures 2B, C**). Based on the existence of a primary branch invasion of the portal vein (either left or right), we separated the cases into two groups: Vp0–Vp2 and Vp3 (**Figures 2D, E**). Analysis of relevant clinicopathological features demonstrated that patients in the Vp3 group had a higher tumor burden. No significant correlation was observed between PVTT status and age, sex, or HBsAg status (**Figure 2F**). Meanwhile, a remarkable trend towards larger tumor dimensions, multiple lesions, and higher levels of AFP and PIVKA-II were observed in the Vp3 group (**Figure 2F**, **Table 1**).

**Figure 2:**
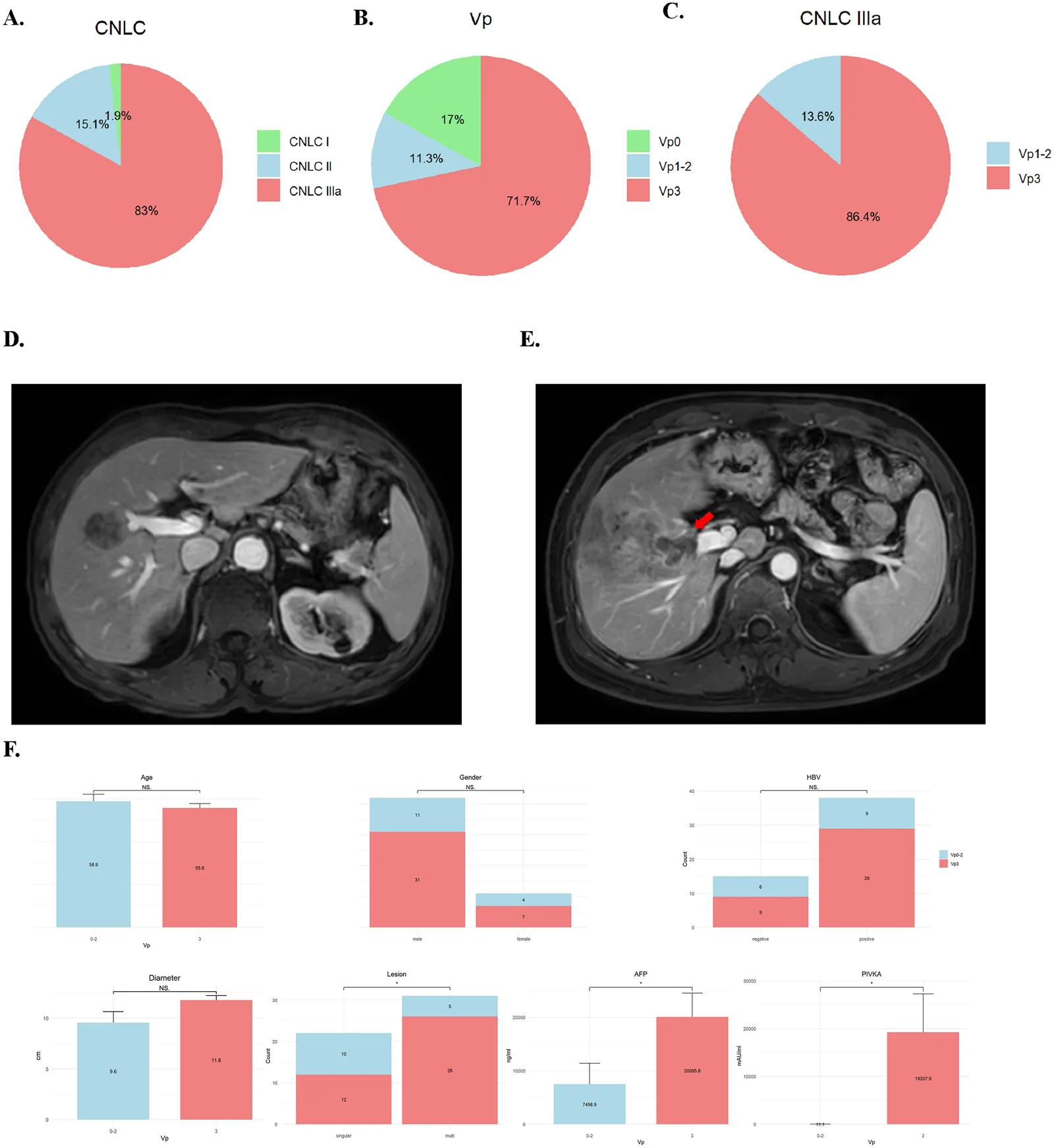
Clinicopathological features of the cohort. **(A)** Proportion of patients stratified by CNLC classification. **(B)** Proportion of patients stratified by Vp classification. **(C)** Proportion of Vp groups within CNLC IIIa group. **(D, E)**. Typical MR images of Vp0 and Vp3 patients. **(F)** Comparison of clinicopathological features between different Vp0–2 and Vp3 groups. Age (58.87 ± 12.75 vs. 55.79 ± 12.10, *p* = 0.430), gender (*p* = 0.708), HBV positive rate (*p* = 0.313) and tumor diameter (9.56 ± 4.23 cm vs. 11.77 ± 2.90 cm, *p* = 0.079) were comparable between Vp0–2 and Vp3 groups. Vp3 group showed significantly higher incidence of multifocal lesions (*p* = 0.030) as well as higher AFP (7498.89 ± 15121.78ng/ml vs. 20095.80 ± 27453.58ng/ml, *p* = 0.039) and PIVKA-II (11.09 ± 8.17mAU/ml vs. 19207.88 ± 49568.58mAU/ml) expression.

**Table 1:** Clinicopathological features.

Variable	Category	Vp0-2	Vp3	p-value
Age		58.87 ± 3.29	55.79 ± 1.96	0.43
Gender	male	11	31	0.708
	female	4	7	
HBsAg	+	9	29	0.313
	–	6	9	
Diameter	(cm)	9.56 ± 1.09	12.29 ± 0.63	0.040^*^
lesions	singular	10	12	0.030^*^
	multi	5	26	
AFP	ng/ml	7498.89 ± 3904.43	20095.80 ± 4453.56	0.039^*^
PIVKA	mAU/ml	11.09 ± 2.18	19207.88 ± 8041.09	0.022^*^

### Vp3 group patients presented enlargement of FLR after 3 therapeutic cycles combined with HAIC

For all 53 unresectable patients, the predicted FLR at baseline was calculated for comparison with later changes. 81.13% cases presented significant tumor control from stable disease (SD) to tumor regression at varying degrees (PR) or complete response (CR). A total of 18.87% of the cases presented with progressive disease (PD), including two cases in the Vp0–2 group and eight cases in the Vp3 group. As shown in **Figure 3A**, regardless of the objective response after three cycles, there was no significant predicted FLR change in the Vp0–2 group cases. In contrast, in the Vp3 group, the FLR change rate showed a potent increase, from 1.37% to 215.03%. Especially, 73.68% (28/38) patients sensitive to the treatment with initial Vp3 PVTT provided a potent enlargement of FLR (from 583.4 ml to 772.8 ml on average, SD = 320.5 and 334.4, *p* < 0.001, **Figures 3A–C**), along with the volume of the opposite hemi liver increasing from 820.0 ml to 928.6 ml on average (SD = 415.1 and 390.3, respectively, *p*<0.0001, **Figures 4A, B**).

**Figure 3:**
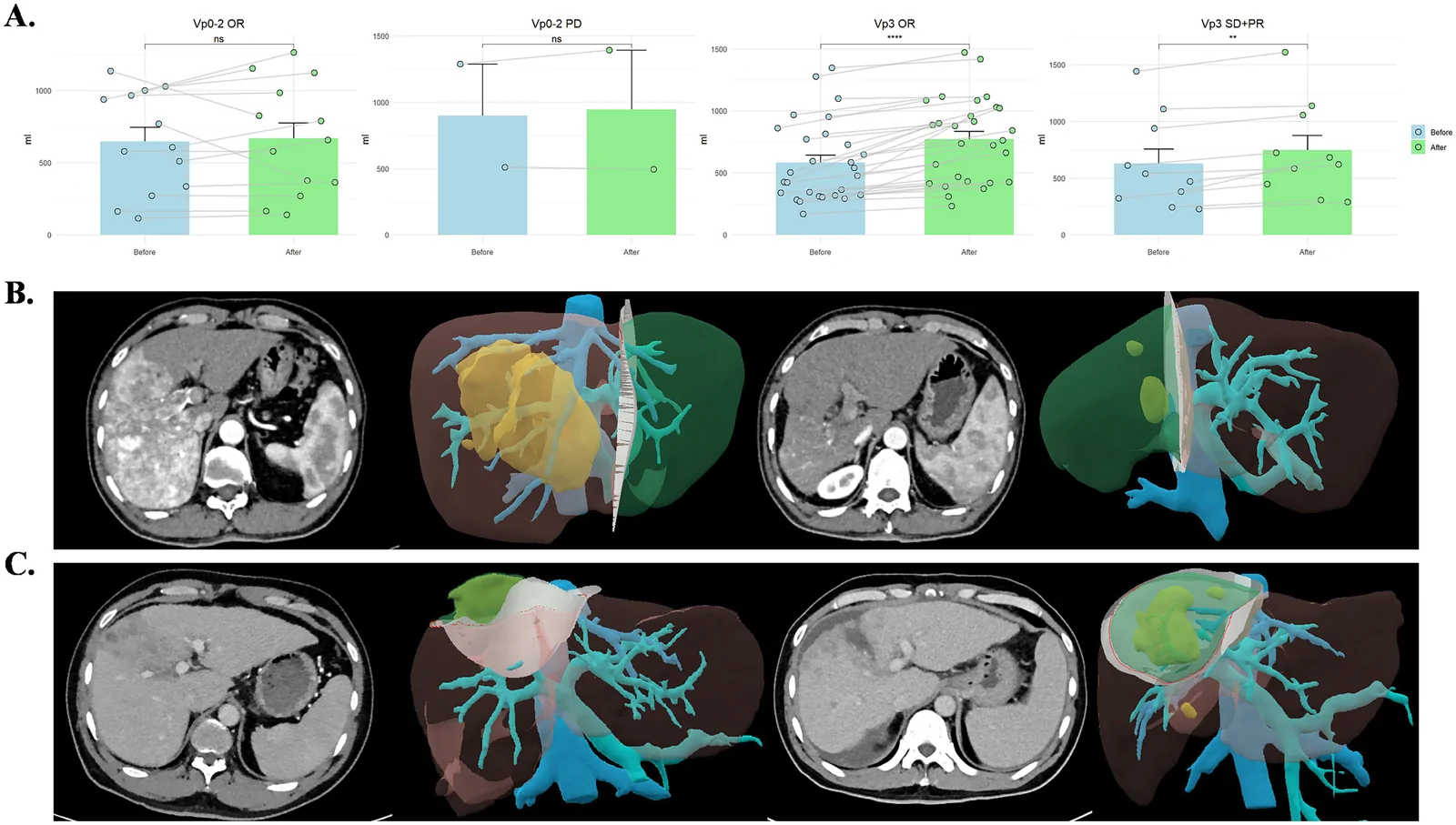
Comparison of FLR between different response groups. **(A)** Paired t-test of FLR between Vp0–2 and Vp3 group with or without OR after treatment. FLR didn’t show meaningful changes in Vp0–2 group no matter the response (OR: before 647.152 ± 352.485ml vs. after 667.668 ± 388.411ml, *p* = 0.701; PD: before 899.155 ± 551.324ml vs. after 944.429 ± 634.799ml, *p* = 0.584). Meanwhile, Vp3 group showed significant FLR increase in both response groups (OR: before 583.425 ± 320.534ml vs. 772.757 ± 334.350ml, *p*<0.001; SD+PD: before 628.693 ± 406.305ml vs. 746.232 ± 412.756ml, *p* = 0.001). **(B, C)**. Typical CT images and rebuild models demonstrating the difference between FLR change in Vp3 **(B)** and Vp0 **(C)** patients.

**Figure 4:**
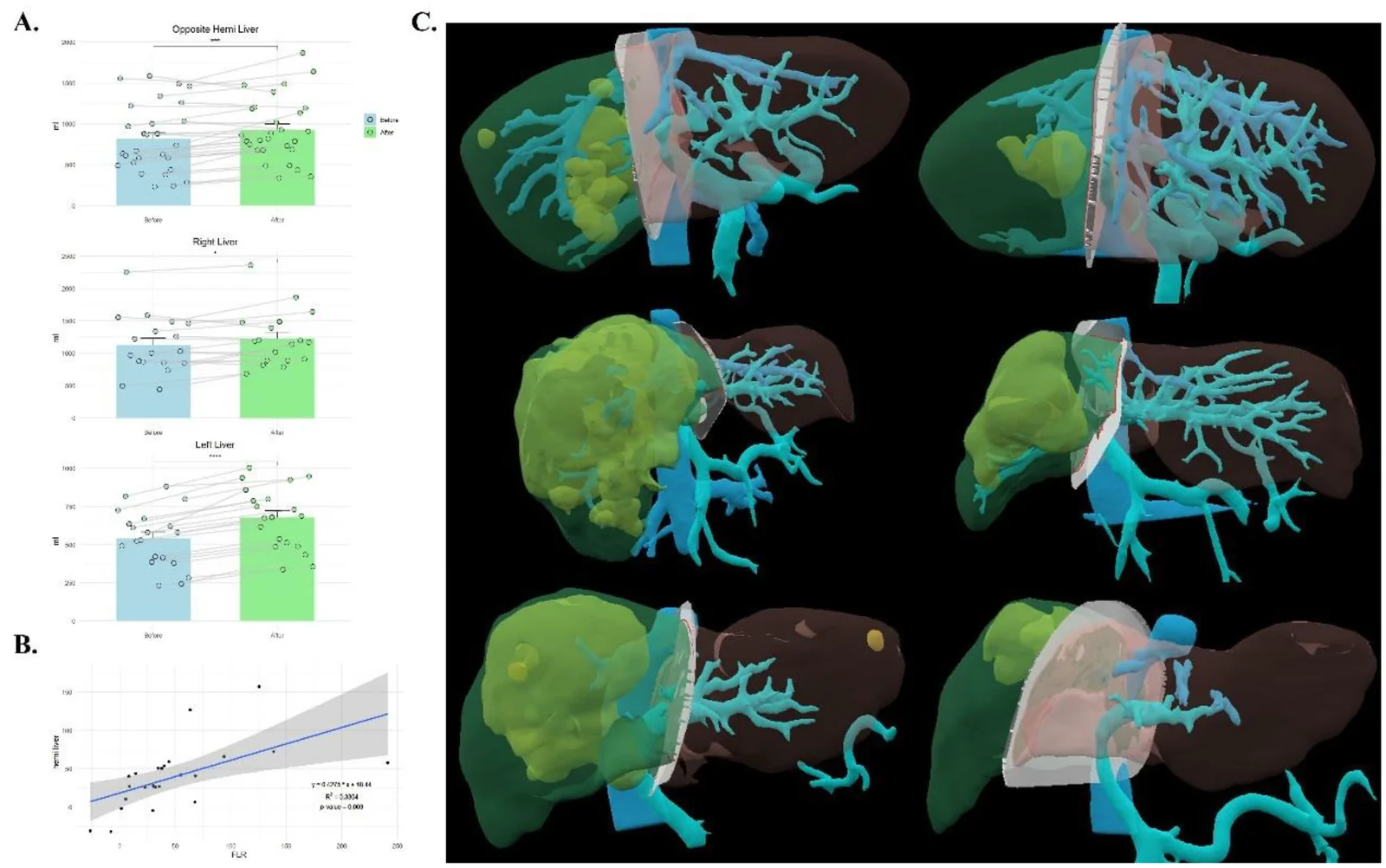
Increase of FLR is related with the enlargement of opposite hemi liver. **(A)** Paired t-test showing the volume changes of hemi livers opposite to the thrombus in Vp3 group with OR. The total volume change is 819.960 ± 415.150ml vs. 928.600 ± 390.306ml, *p* < 0.001. Significant volume increase was found in both sides (right: 1127.211 ± 441.826ml vs. 1227.606 ± 424.675ml, *p* = 0.031; left: 541.53 ± 187.286ml vs. 677.711 ± 207.913ml, *p* < 0.001). **(B)** FLR increase is correlated with enlargement of opposite hemi liver (R^2^ = 0.3304, *p* = 0.003). **(C)** Typical rebuild models illustrating the enlargement of opposite hemi liver induced by HAIC treatment.

We compared the FLR enlargement rate based on whether the primary portal vein Vp3 invasion was on the right or the left side. Typically, the right branch PVTT cases presented an expected significant enlargement of the left liver (FLR changes from 541.5 ml to 677.7 ml, SD = 187.3 and 201.9, respectively, *p* < 0.001, **Figures 4A, C**). Interestingly, the right hemi livers of the cases with the left branch Vp3 invasion, on the other hand, also showed significance in the volume enlargement (FLR changes from 1127.2 ml to 1227.6 ml, SD = 441.8 and 424.7, respectively, *p* = 0.031, **Figure 4A**). These findings strongly support our hypothesis that Vp3-type invasion has a potential advantage in conversion treatment in increasing liver function as a physiological compensatory.

### High necrosis rates of tumor and PVTT were observed in the Vp3 group patients who underwent surgical resection along with the FLR enlargement

Among the 41 patients with an objective response (OR), 15 underwent surgical resection after detailed MDT consultation and evaluation, including 5 Vp0–2 patients and 10 Vp3 patients. FLR increase rate before surgery were 8.7% and 18.5% in Vp0 ~ 2 and Vp3 patients, respectively. The necrosis rate of all specimens was evaluated in both the tumor and the invaded portal vein.

In the Vp3 group, pathological CR (pCR) was determined in one patient, and PR was determined in 27 patients (**Figure 5A**). The Vp3 group had necrosis rates ranging from 78.19% to 100% (average 87.37%, SD = 8.3) for tumors and from 51.74% to 100% (average 83.36%, SD = 17.34) for PVTT. Notably higher than the necrosis rates of the Vp2 cases (from 50.08% to 90.29%, average 73.73%, SD = 13.33 for tumor, *p* = 0.039; 12.34% to 75.21%, average 29.95%, SD = 26.14 for PVTT, *p* = 0.006) (**Figure 5B**). Compared to the Vp0–2 group, the Vp3 cases presented a greater inclination of tumor and thrombus necrosis, and these two indices were positively correlated with the FLR change rate respectively with significance (**Figure 5B**).

**Figure 5:**
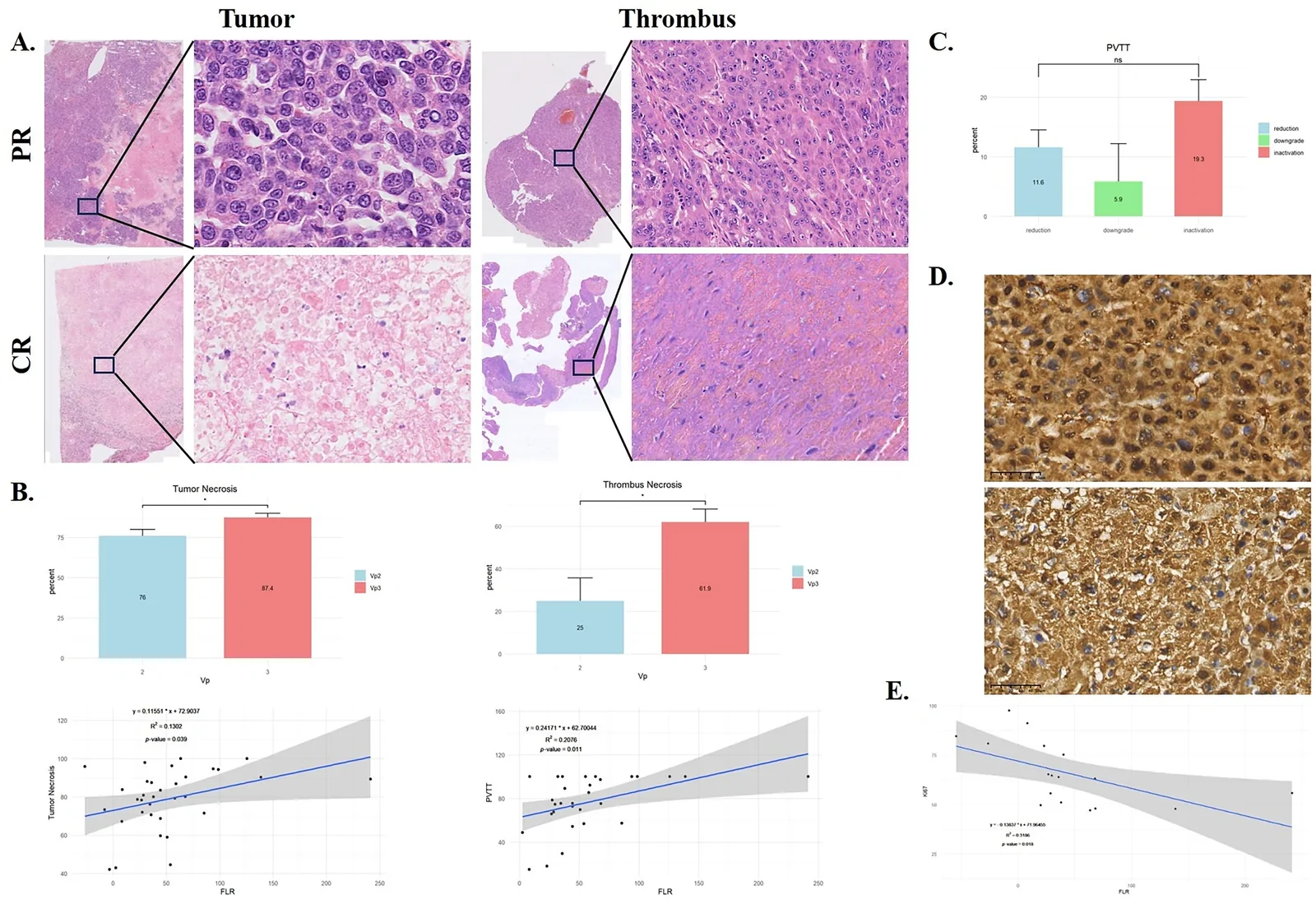
FLR increase indicates tumor and thrombus necrosis and Ki67 expression. **(A)** Typical images of HE-stained tumor samples from PR and CR patients. **(B)** FLR is correlated with tumor and thrombus necrosis. Vp2 and Vp3 groups showed significantly different necrosis rate of tumor (75.952 ± 4.042% vs. 87.366 ± 2.625%, *p* = 0.047) and thrombus (24.961 ± 10.772% vs. 61.934 ± 6.158%, *p* = 0.006). All patients showed significant correlation between FLR increase and tumor or thrombus necrosis (R^2^ = 0.1302 and 0.2076, *p* = 0.039 and 0.011, respectively). **(C)** FLR increasement was not significantly differed between different types of thrombus regression (reduction: 11.585 ± 8.278%, downgrade: 5.853 ± 14.223%, inactivation: 19.345 ± 13.992%, *p* = 0.106). **(D)** Typical IHC images showing differential Ki67 expression in HCC tumor. **(E)** Ki67 expression is negatively correlated with FLR increasement (R^2^ = 0.3189, *p* = 0.018).

### Enlargement of FLR in Vp3 patients correlates with the regression of PVTT

Two main types of PVTT regression were observed in Vp3 patients with OR. 28.57% (8/28) presented a reduction in the tumor thrombus in the cavity of the primary branch, and 17.86% (5/28) were downgraded to Vp2. Simultaneously, 53.57% (15/28) of cases did not show a reduction intra-cavity, but presented a significant inactivation of the tumor thrombus according to MRI and postoperative pathological examination. Interestingly, regardless of the type of PVTT regression, enlargement of the FLR showed no significant difference (**Figure 3C**). Simultaneously, the Ki67 percentage in the specimens was reduced and correlated with FLR changes (**Figures 5D, E**). This means that the change in the opposite hemiliver volume is a synchronistic increase along with the objective response of the conversion therapy.

## Discussion

Based on the anatomical characteristics of the liver and the aggressive features of HCC, the portal vein is the most common vascular system invaded during tumorigenesis (13). The incidence of PVTT in various types is as high as 44%-62.2% in Chinese patients with HCC at diagnosis (14). As is well known, severe PVTT is a thorny symptom that plays a major role in the clinical staging of HCC, and leads to a dismal prognosis and shortened overall survival. Briefly, PVTT develops rapidly, which results in serious portal hypertension, jaundice, and intractable ascites. The median survival of patients with HCC with main portal vein tumor thrombus is 2.7 months (15).

To date, there has been no unified guideline or consensus worldwide for PVTT treatment in advanced HCC, with treatment recommendations differ substantially between Eastern and Western guidelines. The BCLC system is mostly applied by European and American medical centers, and HCC patients with any PVTT type are regarded as BCLC stage C and are recommended to receive systemic therapy (16). Locoregional treatments such as HAIC are not routinely recommended in Western guidelines and remain uncommon in Western clinical practice.

In contrast, in China and several East Asian countries with more complex liver contexts, such as multiple HCC incidences, prevalent chronic hepatitis, and refractory liver dysfunction, newly discovered HCCs are predominantly advanced and inoperable (70%) (11). Thus, more aggressive therapies have been recommended for curative attempts. HAIC has been incorporated into multidisciplinary treatment strategies for selected patients with intermediate-to-advanced HCC, particularly those with macrovascular invasion (17). In the last few years, accumulating clinical studies have showed that a combination of systemic therapy and LRT provides a better synergistic effect on tumor response to induce a higher conversion rate in patients (18). Therefore, the Chinese guidelines support the use of systemic therapy combined with locoregional therapy, including HAIC, as a conversion strategy (19). As demonstrated by our findings, HAIC combined with systemic therapy showed noticeable conversion rate for HCC patients with PVTT, confirming the effectiveness of such treatment.

Severe PVTT reaching the Vp3-type is regarded as one of the most significant risk factors for poor prognosis in advanced HCC characterized by a heavier tumor burden, including sporadic intrahepatic metastasis and complex anatomical issues, resulting in the main limitations and disadvantages of conversion therapy. Notably, in the real world, Vp0–2 is more suitable for curative resection than Vp3–4, with more ideal outcomes. As for Vp3 patients, surgeons not only have to remove a larger scope of segments of the liver, but also need to resect the invaded primary portal branch, and sometimes have to reconstruct the main trunk of the portal vein. However, the operative procedure offers only limited improvement in prognosis, as observed previously (20, 21). Thus, controlling and regressing tumor thrombus in the primary portal vein trunk is also an important objective of conversion therapy. According to a large-scale retrospective study, HAIC has been shown to control tumor progression in advanced HCC patients with macrovascular invasion (22).

For converted advanced HCC patients, another issue is the surgical strategy for Vp3-type patients after responding to treatment. Technical advances in surgery have made hemi-hepatectomy no longer a high-difficulty surgery in most experienced medical centers. However, up to 5–32% of patients may experience post-hepatectomy liver failure (PHLF), which results in almost 50% mortality within 90 days after surgery (23). The exact causes of mortality are inadequate FLR and poor liver function after surgery. Thus, accurate evaluation of FLR is a pivotal perioperative procedure. When Vp3-type patients respond to conversion therapy, inadequate remnant liver function may be an obstacle and a contraindication to undergoing curative surgery.

Enlargement of the remnant liver is an important intervention to create opportunities for patients with an inadequate FLR. Mechanistically, normal hepatocytes do not engage in active cytokinesis during large-scale liver regeneration. However, dramatic division and proliferation occur because of hepatic injury, including acute or chronic inciting events. This is a complex process involving the rapid release of numerous growth factors in the intra-portal vein system and the activity of a large number of signaling pathways (24). For instance, our current basic research revealed that the polyploid status of hepatocytes correlates with cytokinesis and cell proliferation (25, 26). This discovery is also consistent with an intensive study by Zhu et al., which documented the genetic and cellular mechanisms of liver regeneration concerning hepatocyte composition (27, 28). Based on compensatory hypertrophy after portal vein occlusion, a series of strategies to enlarge the FLR has been applied, including the widely used PVE and Associating Liver Partition and Portal Vein Ligation for Staged Hepatectomy (ALPPS) (29). In the real world, preoperative PVE seems much more accepted because of its less invasive nature and greater clinical safety than ALPPS but also meets the limitations of increasing FLR and a relatively longer period for awaiting liver regeneration (30). On the other hand, ALPPS has been reported to be combined with TACE and generated significant hypertrophy (31). However, enlargement effects on the remnant liver depend on uninjured hepatocytes, which limits its effect on liver regeneration in patients with chronic liver diseases or after anti-tumor treatments (32).

Considering in the Vp3-type HCC patients, one of the primary portal vein branches is invaded, we wondered if the tumor thrombus might exert a kind of ‘side effect’ simulating artificial embolization for FLR enlargement during the conversion therapy combined with HAIC. The reasons for proposing this view are twofold. First, anatomically, a Vp3 thrombus indicates advanced tumor progression, an obstacle on one side of the main portal vein branches (either left or right), which is responsible for a remarkable reduction in the portal vein supply to the tumor-side hemi liver. Hepatocyte status and liver function were significantly impaired. Thus, the ipsilateral hemi-liver barely has the opportunity for potential enlargement, especially under systemic therapies and a precisely selected HAIC background, which might have a greater impact on the ipsilateral hemi-liver. Second, on most occasions, the Vp3 thrombus does not impact the blood flow from the main trunk to supply the opposite hemi-liver. HAIC impairment on the opposite side of the liver is limited and controllable. Based on the effect of our conversion therapy, most tumor thrombi can regress to varying degrees. Importantly, even if the blood supply to the tumor side recovers to some extent, a more significant advantage in regeneration and growth is gained by the opposite side of the liver. We speculated that there would be a sufficient time window for the opposite hemiliver to regenerate and hyperplasia during the conversion treatment, similar to the necessary period after PVE.

However, after thoroughly reviewing the literature, we found that the related reports were quite limited, and there was no evidence to validate the contribution of remnant liver enlargement correlated with tumor invasion status, such as PVTT. Moreover, the tumor obstacle caused by PVTT is quite different from the artificial obstacle caused by PVE, and we should consider the tumor’s biological behavior and the probable risk of the abscised tumor cells from the thrombus flowing into the circulation. Thus, exact control and regression of the tumor are the most important prerequisites for establishing any speculation. Another question is whether PVE is normally conducted on the right lobe of the liver to enlarge the left lobe (33), whereas PVTT will not give surgeons the option to set the primary tumor location, and if a Vp3 thrombus occurs on the left, would it affect the right lobe of the liver?

We investigated 53 cases of advanced HCC according to data from our medical center. The common feature of these cases is the strategy for conversion treatment according to the atezo/bev schedule combined with LRT by HAIC. As we mentioned in the ‘Results’ section, the objective response to this conversion therapy is almost 77.36%, which is relatively ideal comparing with the current data in the real world. We focused on cases with Vp3 PVTT, and surprisingly observed widespread hyperplasia of the opposite liver lobe on MRI. The remnant liver volume estimated after 3 therapeutic cycles showed a potent increase in the opposite liver lobe. Notably, remnant liver enlargement occurred with no significant difference among the Vp3-type HCC patients, regardless of the side of portal vein invasion. Similar to PVE, right Vp3-type induced a remarkable increase in the left lobe of the liver. Correspondingly, the left Vp3-type cases also demonstrated a significant increase in the right liver lobe enlargement.

It is worth noticing that larger baseline tumor size and higher tumor burden in Vp3 patients may represent important confounders in our cohort. Regression of the larger tumors may also contribute to the observed FLR increase in Vp3 patients. It is possible that the FLR increase and correlated opposite liver lobe enlargement in Vp3 patients may result from combined mechanism including altered portal perfusion caused by PVTT-related portal venous obstruction, relief of local tissue compression and altered hepatic hemodynamics during massive tumor necrosis. It is important for further study to closely examine these mechanisms in a larger, stratified cohort.

Notably, significant liver enlargement was only observed in Vp3-type patients, and neither the Vp0 nor the Vp1–2 types showed meaningful changes after conversion therapy. We believe that this is a pivotal point in the stratification of advanced patients to design personalized conversion strategies. For Vp0∼2 type patients, the portal vein supply for the ipsilateral hemiliver may partly contribute to the maintenance of hepatocyte function, despite chronic liver diseases such as fibrosis, cirrhosis, or hepatitis. This means that compensatory hepatocyte regeneration and hyperplasia on the opposite side will be weakened. It is also likely that for the Vp4-type patients, a higher incidence of complications such as ascites and liver failure with unsatisfactory PS scores lead to passive situations for conducting intervention treatment, and even if the ICIs and anti-VEGF drug schedules may cause severe adverse events. All these disadvantages require patients to spend much more time to adjust liver function and subside ascites while facing a higher risk of drug resistance or discontinuation (7). Simultaneously, main trunk obstruction of the portal vein strongly limits the application of LRT to preserve essential liver function. Thus, the Vp4-type may benefit less from liver hyperplasia.

Our findings are particularly interesting because Vp0 ~ 2 tumors are generally considered less advanced and more responsive to treatment. However, this apparent susceptibility does not necessarily contradict less FLR enlargement observed in Vp0 ~ 2 patients. In Vp0 ~ 2 patients, portal venous inflow to both lobes is relatively preserved. Therefore, tumor control may be established independent to the compensatory growth of the opposite lobe, which may be more common in Vp3 patients. It is also worth noticing that Vp3 patients’ higher tumor necrosis rate in our study was observed in converted patients. This result cannot be interpreted as greater treatment sensitivity in Vp3 patients, as only selected Vp3 patients with favorable treatment responses were considered for surgery, which may cause selection bias.

Along with the enlargement of the remnant liver, we also noticed a correlation between lower Ki67 expression in tumor tissues after conversion therapy. Since Ki67 significantly indicates the cell proliferation capacity of tumor cells (34), it can be easily understood that when the Vp3-type patients objectively responded to the conversion therapy, the cell proliferation from the tumor side hemi liver was remarkably impaired by combining HAIC therapy, which should be a pivotal stimulation for liver hyperplasia on the opposite side.

In summary, as is commonly acknowledged, Vp3-type HCC is always regarded as an advanced tumor stage, with a series of disadvantages for conversion. However, based on the specific anatomical characteristics, the pathological obstacle on the ipsilateral portal vein potentially simulates artificial embolization for liver enlargement. During conversion therapy, patients may simultaneously experience hepatocyte regeneration and hyperplasia in the opposite lobe of the liver. Thus, advanced HCC patients with Vp3 invasion on the tumor side may be stratified independently as a population with potential benefits of the enlargement of FLR and safe resection after an objective response to the conversion therapy.

## Data Availability

The original contributions presented in the study are included in the article/supplementary material. Further inquiries can be directed to the corresponding authors.
